# Common Hepatic Artery Resection and Reconstruction During Pancreaticoduodenectomy Following Chemotherapy for Locally-Advanced Pancreatic Cancer: A Case Report

**DOI:** 10.7759/cureus.94956

**Published:** 2025-10-19

**Authors:** Tomohiro Nakajima, Yutaka Iba, Tsuyoshi Shibata, Shigeki Komatsu, Masafumi Imamura

**Affiliations:** 1 Department of Surgery, Division of Cardiovascular Surgery, Sapporo Medical University, Sapporo, JPN; 2 Department of Surgery, Division of Gastroenterological Surgery, Sapporo Medical University, Sapporo, JPN

**Keywords:** arterial invasion, cancer of the pancreas, combined resection, common hepatic artery, pancreatic cancer, vascular reconstruction

## Abstract

A 73-year-old female patient was diagnosed with unresectable locally advanced pancreatic cancer at 72 years of age and underwent chemotherapy. Due to a successful response to chemotherapy, she was scheduled for a pancreaticoduodenectomy and common hepatic artery resection by the gastrointestinal surgery department. As the common hepatic artery was to be resected, our cardiovascular surgery department was consulted for vascular reconstruction to ensure hepatic blood flow. During the surgery, our team intervened prior to tumor resection. The pancreaticoduodenal artery was infiltrated by the tumor and required resection. Although the common hepatic artery proximal to its branches had to be resected, we determined that a direct end-to-end anastomosis between the remaining common hepatic artery and the proper hepatic artery was feasible. To maintain a wide anastomotic opening, both ends were trimmed obliquely. An end-to-end anastomosis was performed using 6-0 PROLENE® Polypropylene Suture (Ethicon, Johnson & Johnson, Somerville, NJ, USA). Blood flow was confirmed to be adequate using a flow meter. Subsequently, the gastrointestinal surgery department proceeded with tumor resection. The patient had an uneventful postoperative course and was discharged on postoperative day 22. This case highlights that prompt, direct hepatic arterial reconstruction can safely preserve hepatic inflow without the need for interposition grafting.

## Introduction

Pancreatic ductal adenocarcinoma (PDAC) often presents as a disease not immediately amenable to resection, yet modern multi-agent chemotherapy, exemplified by FOLFIRINOX (folinic acid, fluorouracil, irinotecan, and oxaliplatin), has improved systemic control and helped establish the concept of conversion surgery, in which initially unresectable tumors become candidates for resection after treatment response [1-3].

Within this evolving paradigm, vascular procedures may be required in selected cases. Notably, arterial resection and reconstruction remain uncommon and technically demanding and are associated with higher procedure-related risks according to systematic reviews and meta-analyses [4,5]. These data underscore the importance of careful patient selection, meticulous planning, and multidisciplinary collaboration when arterial involvement is suspected.

Against this background, we report a case of pancreaticoduodenectomy with common hepatic artery resection and end-to-end reconstruction performed after favorable response to neoadjuvant chemotherapy, highlighting selection criteria, technical considerations, and coordinated management by gastrointestinal and vascular surgery teams.

## Case presentation

**Figure 1:**
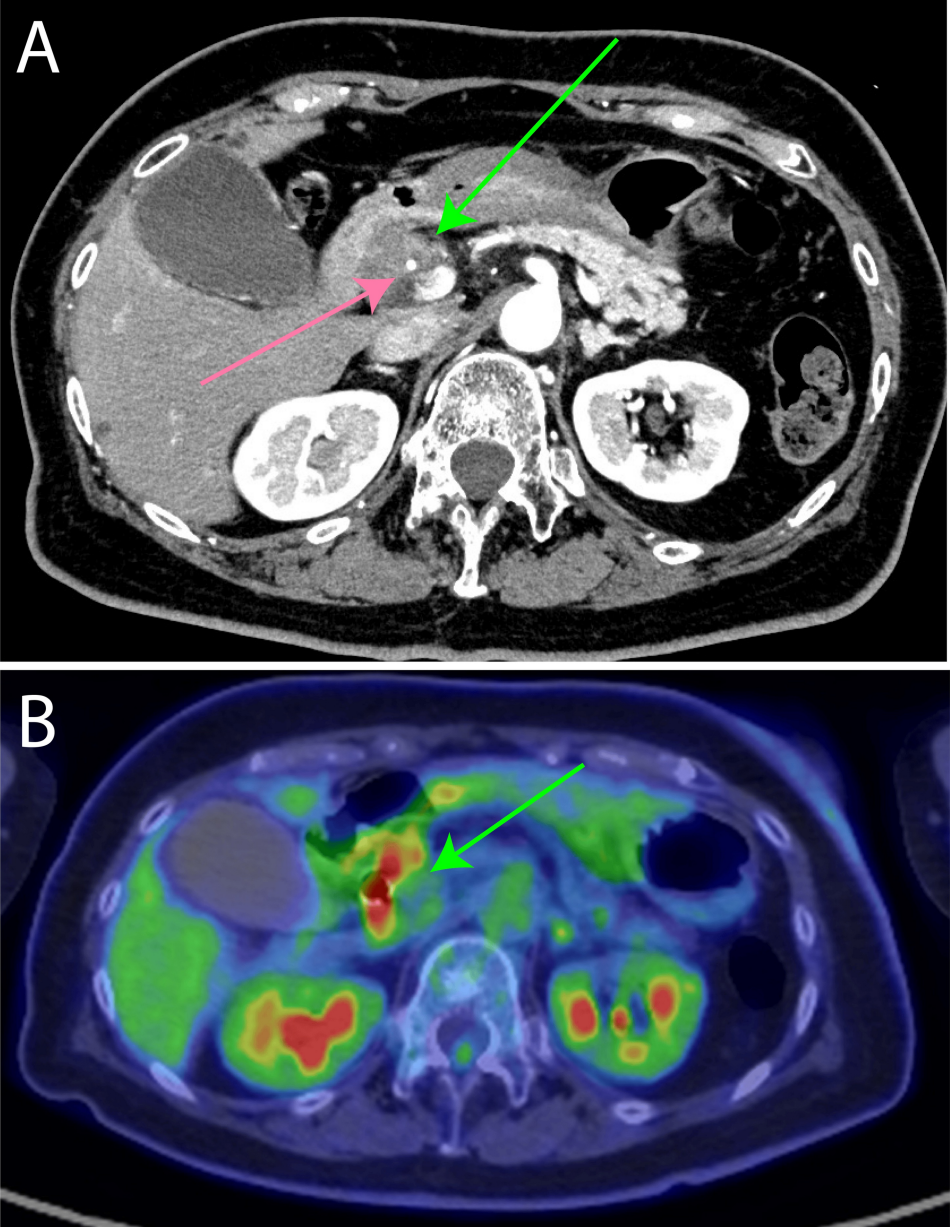
Images at the time of diagnosis of pancreatic cancer (patient's age: 72 years) (A) Contrast-enhanced CT showing a pancreatic head tumor (green arrow) with the gastroduodenal artery identified within the tumor (pink arrow); (B) Positron Emission Tomography-Computed Tomography (PET-CT) demonstrating radiotracer uptake corresponding to the tumor (green arrow).

A 73-year-old woman was diagnosed with pancreatic duct dilation on abdominal echocardiography at the age of 72 years (Figure 1).

**Figure 2:**
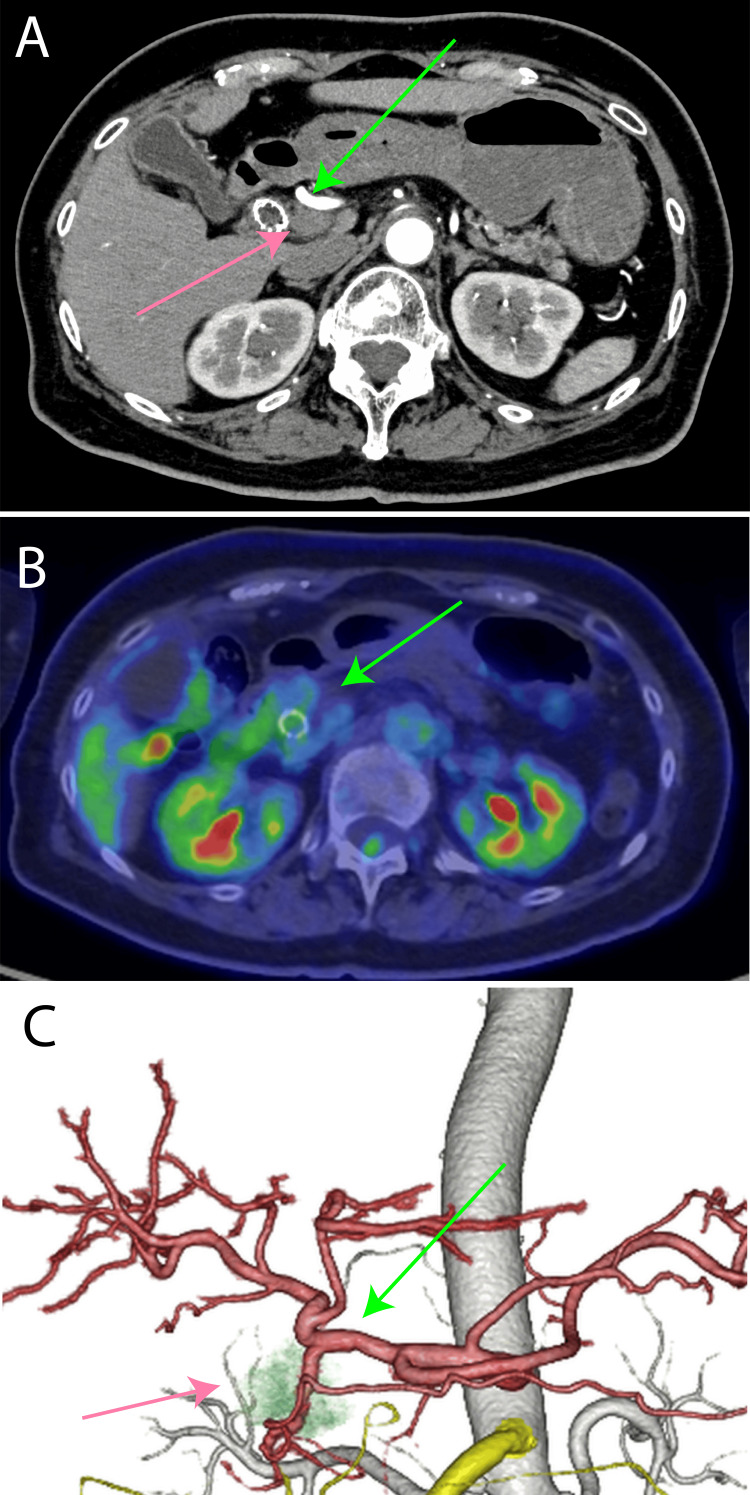
Computed tomography and Positron Emission Tomography-Computed Tomography (PET-CT) scan after chemotherapy (A) Contrast-enhanced CT showing the common hepatic artery (green arrow) and the tumor (pink arrow); (B) PET-CT showing disappearance of the radiotracer uptake previously corresponding to the tumor (green arrow); (C) Volume-rendered (VR) image demonstrating that although the common hepatic artery (green arrow) is in close proximity to the tumor (pink arrow), there is no evidence of vascular invasion.

She was treated with 11 courses of gemcitabine plus nanoparticle albumin-bound paclitaxel (GEM+nab-PTX). The patient's disease was maintained for a long period of time, and the Positron Emission Tomography-Computed Tomography (PET-CT) follow-up scan showed no radiological evidence of tumor. The patient was judged to be suitable for surgery. The tumor had invaded the gastroduodenal artery and its margins extended near the proper hepatic artery (Figure 2).

In addition to pancreaticoduodenectomy, vascular resection near the proper hepatic artery was necessary, and the vascular surgery department was requested to assist. When the tumor extended into the common hepatic artery, we prepared for surgery by performing both direct anastomosis with a nearby vessel and bypass using the great saphenous vein as a free graft as a reconstruction method.

**Figure 3:**
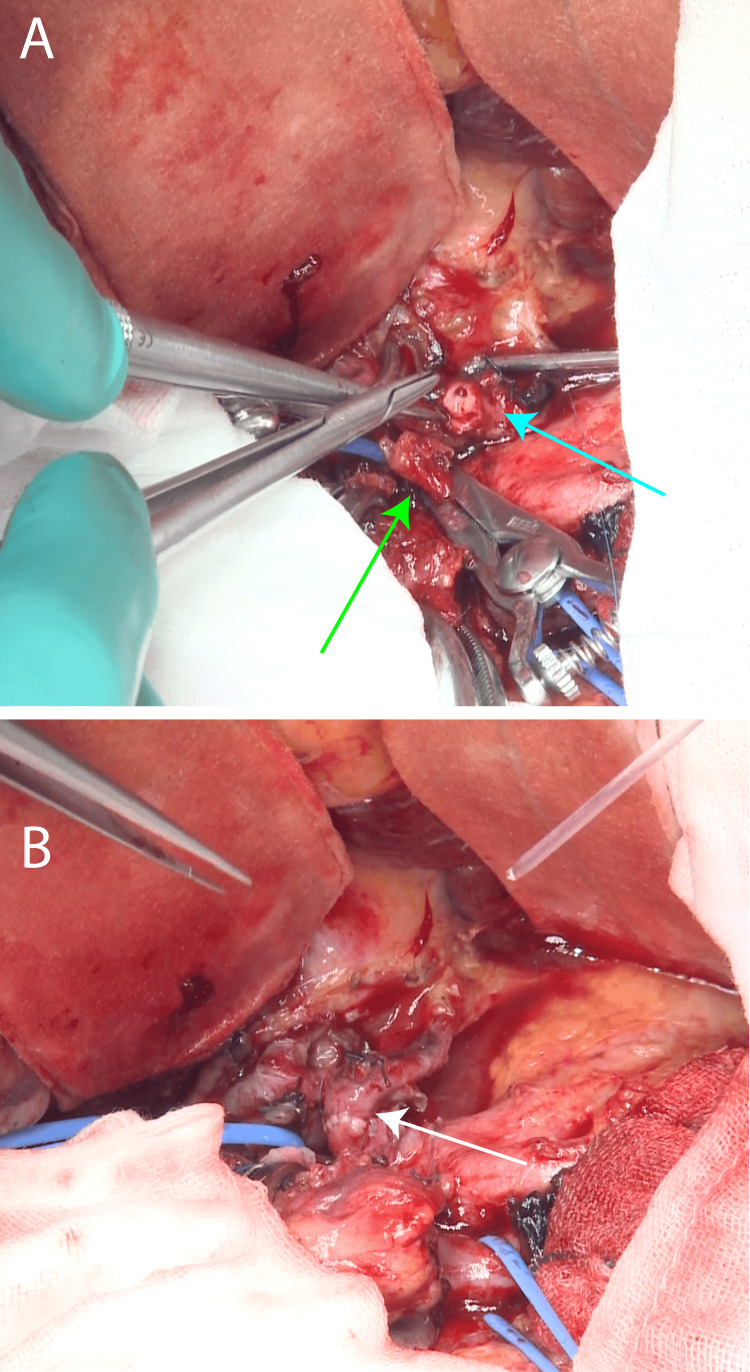
Intraoperative images (A) Image at the time of anastomosis. Common hepatic artery (green arrow) and hepatic artery (blue arrow); (B) Image after anastomosis. Anastomosis site (white arrow).

The patient underwent surgery under general anesthesia. Gastroenterological surgery was performed through a midline abdominal incision. The tumor and pancreatic head were identified. The tumor extended to the vicinity of the bifurcation of the common hepatic artery from the gastroduodenal artery, and it was decided to resect the posterior superior pancreaticoduodenal artery and the tumor as a single lump, followed by anastomosis of the common hepatic artery and the proper hepatic artery. After confirming that the activated clotting time was more than 250 seconds, the common hepatic artery, right hepatic artery, left hepatic artery, and gastroduodenal artery were blocked. A portion of the common hepatic artery, including the gastroduodenal artery, was severed as planned (Figure 3).

**Figure 4:**
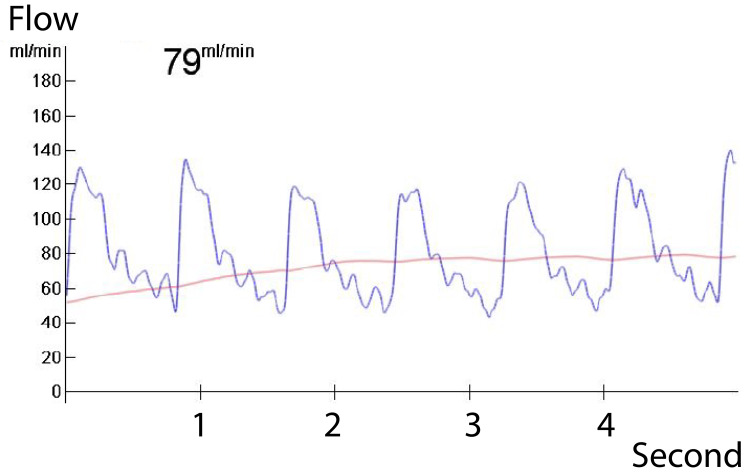
Flow meter report (blood flow of 79 mL/min observed)

The blood flow was checked with a flow meter and was confirmed to be 79 ml/min (Figure 4).

**Figure 5:**
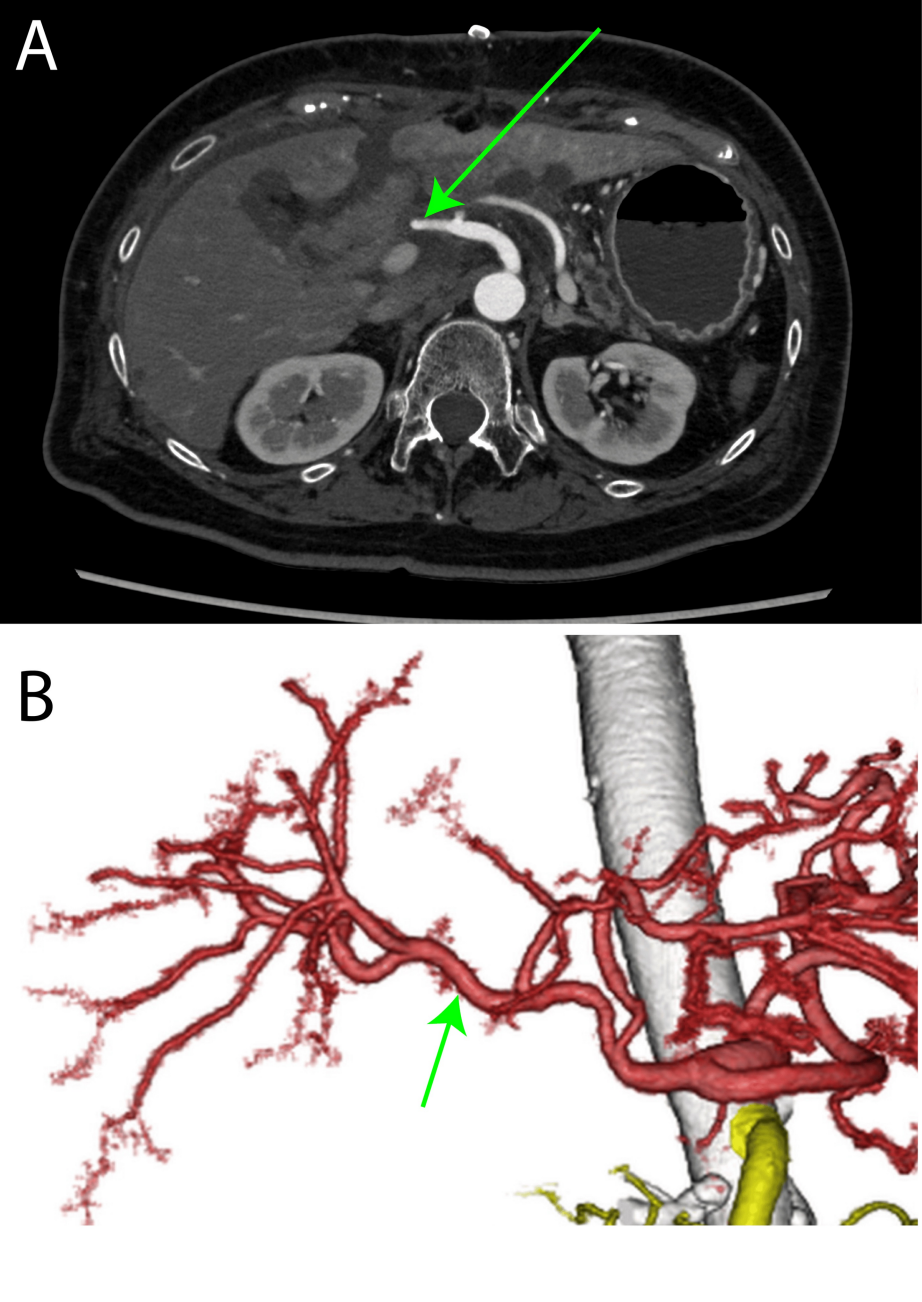
Postoperative CT (A) Contrast-enhanced CT showed the anastomosis site with common hepatic artery and proper hepatic artery (green arrow); (B) Volume-rendered (VR) image demonstrating the anastomosis site with common hepatic artery and proper hepatic artery (green arrow).

Heparin was antagonized with protamine, and the vascular surgical intervention was completed. The tumor was removed and the abdomen was closed. One week postoperatively, contrast-enhanced CT confirmed that there were no problems with the anastomosis or its blood flow (Figure 5).

The patient was discharged home on the 22nd postoperative day. At the three-month follow-up visit, the patient was alive and clinically well.

## Discussion

PDAC with arterial involvement has traditionally been considered a contraindication for surgical resection due to the complexity and high risk of complications associated with arterial reconstruction [6]. However, with the advent of more effective chemotherapy regimens, such as gemcitabine with nab-paclitaxel and FOLFIRINOX, the concept of conversion surgery has gained traction [7]. Selected patients with initially unresectable tumors are now candidates for surgery after demonstrating significant tumor regression and no distant metastases [8,9].

Despite these advancements, arterial resection and reconstruction during pancreaticoduodenectomy remain technically demanding and relatively rare. Portal vein resection has become increasingly standardized in high-volume centers, but hepatic artery reconstruction, especially involving the common hepatic artery, is less frequently performed due to its anatomical complexity and risk of postoperative ischemic complications. In this context, multidisciplinary collaboration, including vascular surgery expertise, is crucial to ensure oncologic radicality while preserving adequate hepatic perfusion.

In the present case, the patient demonstrated a good response to chemotherapy and was deemed a candidate for curative resection. However, the tumor’s proximity to and invasion of the common hepatic artery posed a significant challenge. Our strategy included preoperative planning for both direct end-to-end anastomosis and bypass reconstruction using the great saphenous vein. Intraoperatively, trimming and oblique cutting of the arterial ends allowed for tension-free direct anastomosis, achieving satisfactory hepatic blood flow of 79 ml/min, confirmed via intraoperative flow measurement. Acceptable results were obtained by end-to-end anastomosis without grafting.

Previous studies have reported favorable outcomes in selected patients undergoing hepatic artery reconstruction during pancreatic surgery, although such cases remain limited. Abou-Khalil et al. emphasized that even arterial involvement can be addressed surgically when combined with effective neoadjuvant therapy and meticulous vascular reconstruction techniques [10]. Similarly, Brasoveanu et al. reported that direct reconstruction of the hepatic artery can be safely performed in well-selected cases, with acceptable morbidity and survival outcomes [11].

This case highlights the feasibility of vascular resection and reconstruction for hepatic artery involvement in pancreatic cancer when performed in a multidisciplinary setting. It underscores the importance of collaboration between gastrointestinal and vascular surgeons in expanding the indications for curative surgery in pancreatic cancer.

## Conclusions

Hepatic artery reconstruction during pancreaticoduodenectomy can be performed safely and effectively in selected cases of locally advanced pancreatic cancer, particularly after a favorable response to neoadjuvant chemotherapy. Meticulous preoperative planning, intraoperative adaptability, and close collaboration between gastrointestinal and vascular surgeons are essential to achieve oncologic radicality while preserving hepatic perfusion. As conversion surgery becomes increasingly feasible, the role of vascular surgeons is likely to expand in carefully chosen cases requiring arterial resection and reconstruction.
